# Impact of gender on left atrial low-voltage zones in patients with persistent atrial fibrillation: results of a voltage-guided ablation

**DOI:** 10.3389/fcvm.2023.1229345

**Published:** 2023-08-24

**Authors:** Halim Marzak, Romain Ringele, Kensuke Matsushita, Benjamin Marchandot, Simon Fitouchi, Thomas Cardi, Mohamad Kanso, Alexandre Schatz, Justine Hammann, Patrick Ohlmann, Olivier Morel, Laurence Jesel

**Affiliations:** ^1^ Division of Cardiovascular Medicine, Nouvel Hôpital Civil, Strasbourg University Hospital, Strasbourg, France; ^2^ Regenerative Nanomedicine, Faculty of Pharmacy, UMR 1260 INSERM (French National Institute of Health and Medical Research), University of Strasbourg, Strasbourg, France

**Keywords:** atrial fibrillation, gender, low-voltage zones, atrial bipolar voltage, voltage-guided ablation

## Abstract

**Background:**

Gender-related differences have been reported in atrial fibrotic remodeling and prognosis of atrial fibrillation (AF) patients after ablation. We assessed in persistent AF the regional distribution of left atrial (LA) bipolar voltage and the extent of low-voltage zones (LVZ) according to gender as well as the results of a voltage-guided substrate ablation.

**Methods:**

Consecutive patients who underwent a voltage-guided AF ablation were enrolled. LA endocardial voltage maps were obtained using a 3D electro-anatomical mapping system in sinus rhythm. LVZ was defined as <0.5 mV.

**Results:**

A total of 115 patients were enrolled (74 men, 41 women). The LA bipolar voltage amplitude was twice lower in the whole LA (*p* < 0.01) and in each atrial region in women compared with men, whereas the LA indexed volume was similar. LVZ were found in 56.1% of women and 16.2% of men (*p* < 0.01). LVZ were also more extensive in women (*p* = 0.01), especially in the anterior LA. Atrial voltage alteration occurred earlier in women than in men. In a multivariate analysis, the female sex (OR 12.99; 95% CI, 3.23–51.63, *p* = 0.0001) and LA indexed volume (OR 1.09; 95% CI, 1.04–1.16, *p* = 0.001) were predictive of LVZ. Atrial arrhythmia-free survival was similar in men and women 36 months after a single ablation procedure.

**Conclusion:**

The study reports a strong relationship between the female gender and atrial substrate remodeling. The female gender was significantly associated with higher incidence, earlier occurrence, and greater extent of LVZ compared with men. Despite the female-specific characteristics in atrial remodeling, LVZ-guided ablation may improve the AF ablation outcome in women.

## 1. Introduction

Gender-related differences in atrial fibrillation (AF) are an important issue, which are underappreciated, compared with coronary artery disease, heart failure, or stroke.

Recent studies have demonstrated sex-related differences in the incidence, clinical characteristics, and prognosis of AF patients (1–3).

While AF incidence remains low in adults <50 years of age in both sex, its age-adjusted incidence is estimated to be 1.5–2 times lower in women than in men (1). In general, women develop AF a decade later than men, are more symptomatic, and are more likely to present with atypical symptoms contributing to delayed diagnosis and care (2). Moreover, sex-related differences have been reported in atrial fibrotic remodeling (4–6). Higher atrial fibrosis assessed by late-gadolinium enhancement magnetic resonance imaging (LGE-MRI) was observed among women with AF (4) and associated with AF recurrence (5, 6).

Recently, left atrial low-voltage zones (LVZ) assessed by electro-anatomical mapping (EAM) were determined as a surrogate marker of atrial fibrosis (7) and correlated with areas presenting delayed gadolinium enhancement on MRI (4–6, 8).

LVZ are a powerful predictor of recurrence after AF catheter ablation (CA) (9). The association between LVZ and female sex has been mentioned in several studies (10, 11). To date, scarce data exist about the relationship between LVZ and left atrial (LA) bipolar voltage comparing men and women (3, 11, 12). Pulmonary vein isolation (PVI) in combination with LVZ-guided ablation could provide better results in persistent AF associated with long-term 50% recurrence of atrial arrhythmia (AA) (13–16). There are no data regarding the outcome after LVZ-guided CA according to gender in persistent AF.

To fill this knowledge gap, we aimed both in an unmatched and matched cohort of persistent AF patients referred to our institution for CA to assess the LVZ extent and the regional distribution of LA bipolar voltage according to gender. The other goal was to evaluate the outcome 36 months after LA voltage-guided substrate ablation in addition to PVI.

## 2. Methods

### 2.1. Study population

This observational study enrolled 281 patients who underwent initial ablation of persistent AF at Strasbourg University Hospital, France, between November 2017 and December 2019.

After exclusion of patients without LA voltage maps in sinus rhythm (SR), 166 patients were enrolled. A total of 51 patients with a structural heart disease were also excluded (Supplementary Figure S1). A structural heart disease was defined as ischemic heart disease, valve dysfunction (≥ moderate), or primary myocardial structural disease including dilated cardiomyopathy and hypertrophic cardiomyopathy. Finally, 115 patients were included in an unmatched cohort.

The patient demographics and baseline clinical characteristics were recorded.

Written informed consent for the ablation and the use of data for research purposes was obtained from all patients before the procedure of AF catheter ablation, and the study protocol was approved by the Institutional Review Board of the University of Strasbourg.

### 2.2. Propensity score-matched cohort study

To minimize the confounding factors on the occurrence of LVZ, a propensity score (PS) matching was assessed. The propensity score model included the following covariates: AF duration, time to treatment (time from first clinical diagnosis of AF to ablation procedure), age, body mass index (BMI), CHA_2_DS_2_-VASc* (without female), Hypertension (HT), dyslipidemia, diabetes, obstructive sleep apnea, previous thrombo-embolic events, paroxysmal AF history, and estimated glomerular filtration rate (eGFR). A nearest neighbor algorithm was used to match sex difference in a 1:1 ratio, with a caliper width equal to 0.2 of the standard deviation of the logit of the propensity score. The propensity score-matched cohort included a total of 56 patients with 28 males and 28 females. The patient demographics and baseline clinical characteristics were recorded.

### 2.3. Electro-anatomical voltage mapping

The CA procedures were performed under general anesthesia using a three-dimensional (3D) electro-anatomical mapping system (CARTO 3, Biosense Webster, Diamond Bar, CA, USA, or EnSite Velocity, Abott, St Paul, MN, USA) and a deflectable decapolar circular mapping catheter [Lasso™ NAV catheter, diameter 20 mm, interelectrode spacing 6 mm, Biosense Webster, Diamond Bar, CA, USA, or a spiral multipolar pulmonary vein (PV) catheter, Afocus II, diameter 20 mm, electrode spacing 5 mm, Abott, St Paul, MN, USA]. LA endocardial voltage mapping was performed in SR prior to radiofrequency ablation. SR was achieved with electrical cardioversion in patients with AF.

An endocardial contact during point acquisition was validated by a stable contact signal for >2 beats. All points recorded in SR were analyzed to exclude mechanically induced premature beats. Any area with abnormal voltage resulting from inadequate contact between the circular catheter and LA tissue was reassessed carefully point by point with a 4-mm irrigated contact-force ablation catheter (ThermoCool® SmartTouch®, Biosense Webster, Diamond Bar, CA, USA, or Tacticath®, Abott, St Paul, MN, USA) to avoid any mistakes. LA was divided into six anatomical regions: posterior, anterior, septal, lateral, left atrial appendage (LAA), and inferior (Supplementary Figure S2). The roof was part of the anterior region as previously described (17). A large number of voltage points were collected in each atrial region. A deep mapping of all LAA was consistently made for each patient (Supplementary Figure S3).

The bipolar voltage amplitude was recorded for every point and within each individual region. The median LA (=all regions) and the bipolar voltage measurements of each specific region were calculated.

The left atrial intracavitary volume (LAIV) excluding LAA was obtained for each patient after LA anatomic reconstruction and expressed in ml. The left atrial intracavitary volume index (LAIVI) corresponded to LAIV indexed to the body surface and expressed in ml m^2^.

LVZ was defined as sites of >3 adjacent low-voltage points with a bipolar peak-to-peak voltage amplitude of <0.5 mV (8, 18) and covering >5% of the LA surface area (LVZ surface/LA surface >5% excluding the pulmonary venous antral region, LAA orifice, and mitral valve). This threshold value corresponds to the lowest degree of atrial fibrosis detected using LGE-MRI (6). The LVZ extent was categorized as Stage I (no LVZ, ≤5%), II (mild, >5%–≤20%), III (moderate, >20%–≤35%), and IV (severe, >35%) according to the UTAH fibrosis classification (6). Each region involving LVZ was considered as low-voltage region. The surface areas (cm^2^) of each atrial region and of LVZ within each region were measured using the software of 3D-EAM.

The duration from the onset of *P*-wave to the local activation time of the LAA (P-LAA) was measured after LA voltage mapping for each patient in SR.

### 2.4. Radiofrequency catheter ablation procedure

Ablation was performed with a maximal power limit of 35 W (20–25 W power at the posterior wall and 30–35 W at other areas, infusion rate of 17 ml/min) using an irrigated contact-force catheter. The ablation protocol was the same in the two groups. If the LA voltage map was normal, PVI was only performed for Stage I patients, whereas LVZ homogenization or isolation in addition to PVI was done in patients with mild or moderate LVZ. Linear ablation was done when LVZ could be seen as a critical isthmus site for a potential macro-reentrant tachycardia*.* At the end of CA, an arrhythmia induction was performed by atrial burst pacing to refractoriness. No additional ablation lesions were performed except for an organized atrial tachycardia (AT).

### 2.5. Follow-up

Antiarrhythmic drugs (AADs) were continued in all patients during a blanking period of 3 months after CA during which arrhythmia recurrences were not judged as ablation failure. The patients were reviewed at 3 months, then every 6 months until the 42nd month by their cardiologist. At each time point, a 12-lead ECG and 24-h Holter ECG were recorded. A recurrence of AAs was deﬁned as any documented AF, atrial flutter, and AT lasting more than 30 s with or without AADs. AADs were gradually discontinued between 3 and 6 months post-ablation in the absence of recurrence at the discretion of the physician.

### 2.6. Statistical analysis

The Shapiro–Wilk test was used to determine the Gaussian distribution for each quantitative variable. Normally distributed variables were expressed as mean ± SD. The variables with a non-Gaussian distribution were expressed as median (25th–75th interquartile range). The categorical variables were presented as a number and percentage. The statistical differences of the categorical variables between genders were tested using chi-square test or Fischer's exact test. The differences of quantitative variables were evaluated for statistical significance using Student’s *t*-test or Wilcoxon test, depending on data distribution. One-way ANOVA was applied to assess the significant differences between three or more groups. If significant, Tukey's *post-hoc* test was used to detect the level of significant differences.

The event-free survival was demonstrated using Kaplan–Meier survival curves for men and women using the log-rank test with Bonferroni correction. Binominal logistic regression was used to calculate the odds ratio (OR) and 95% confidence interval (CI) of independent variables associated with LVZ. The variables selected for testing in the multivariate analysis were those with *p* < 0.15 in the univariate analysis. All statistical analyses were performed using SPSS statistical software, version 23.0 (IBM Corp.) for the unmatched cohort. A two-tailed *p-*value of <0.05 was considered statistically significant.

The propensity score model was developed using logistic regression. For the matched cohort, all analyses were performed using R version 3.6.3.

## 3. Results

### 3.1. Baseline characteristics

A total of 115 patients, predominantly male (64%) with initial ablation of persistent AF met the inclusion criteria (Supplementary Figure S1). For the unmatched cohort, the traditional cardiac risk factors were similar between men and women. Women were older [70 (63–73) vs. 63 (56–68) years, *p* < 0.01] and had a mild altered kidney function than men (*p* < 0.01). Lower LA intracavitary volume was observed among women without reaching significance (*p* = 0.06), but no difference regarding indexed values was further observed (*p* = 0.71). The patient characteristics before and after PS matching of the primary cohort are available in Table 1.

**Table 1:** Baseline characteristics according to gender before and after PS matching. *Absence of data because statistical analysis is impossible to perform.

	Before PS matching			PS-matched		
	Men (*n* = 74)	Women (*n* = 41)	*p*-value	Men (*n* = 28)	Women (*n* = 28)	*p*-value
Age, years	62.39 ± 8.98	67 ± 9.46	<0.01	66.71 ± 5.79	67.29 ± 7.70	0.755
BMI, kg/m^2^	29.22 ± 3.55	30.75 ± 7.84	0.560	29 ± 3.46	29.25 ± 6.99	0.893
Dyslipidemia, *n* (%)	25 (33.78)	13 (31.71)	0.98	7 (25.0%)	8 (28.6%)	1.000
Hypertension, *n* (%)	43 (58.11)	27 (65.85)	0.54	18 (64.3%)	19 (67.9%)	1.000
Diabetes mellitus, *n* (%)	11 (14.86)	10 (24.39)	0.31	6 (21.4%)	6 (21.4%)	1.000
Smoking, *n* (%)	14 (18.92)	4 (9.76)	0.3	7 (25.0%)	3 (10.7%)	0.295
OSA, *n* (%)	24 (32.43)	15 (36.59)	0.81	11 (39.3%)	9 (32.1%)	0.781
Coronary artery disease, *n* (%)	13 (17.57)	2 (4.88)	0.1	5 (17.9%)	1 (3.6%)	0.193
Previous thrombo-embolic events, *n* (%)	8 (10.81)	6 (14.63)	0.56	2 (7.1%)	3 (10.7%)	1.000
Paroxysmal AF history, *n* (%)	24 (32.43)	18 (43.9)	0.31	11 (39.3%)	12 (42.9%)	1.000
Sinus node dysfunction, *n* (%)	7 (9.46)	3 (7.32)	1	2 (7.1%)	3 (10.7%)	1.000
Time to treatment, days	675 [182–1,505]	616 [259–2,271]	0.79	708 [199–2,414]	775 [415–2,228]	0.670
Reported AF duration, months			0.46			0.112
<3	57 (78.08%)	32 (78.05%)		19 (67.9%)	20 (71.4%)	
≥3 to <6	10 (13.7%)	4 (9.76%)		6 (21.4%)	3 (10.7)	
≥6 to <9	2 (2.74%)	4 (9.76%)		0 (0.0%)	4 (14.3%)	
≥9 to <12	2 (2.74%)	0 (0%)		2 (7.1%)	0 (0.0%)	
≥12	2 (2.74%)	1 (2.44%)		1 (3.6%)	1 (3.6%)	
CHA_2_DS_2_-VASc score	1.85 ± 1.39	3.14 ± 1.35	<0.01	1.93 ± 1.21	3.11 ± 1.40	0.001
CHA_2_DS_2_-VASc score (without female discrimination)	1.85 ± 1.39	2.14 ± 1.35	0.2	1.93 ± 1.21	2.11 ± 1.40	0.612
Beta blocker	64 (86.49%)	32 (78.05%)	0.37	25 (89.3%)	23 (82.1%)	0.705
ACEi/ARB	51 (68.92%)	22 (53.66%)	0.15	21 (75.0%)	16 (57.1%)	0.259
Aldosterone receptor antagonist	14 (18.92%)	10 (24.39%)	0.65	6 (21.4%)	6 (21.4%)	1.000
*P*-wave duration, ms	124.07 ± 19.18	120.14 ± 31.82	0.25	*	*	*
eGFR, ml/min/1.73 m^2^	90 (69.5–95.75)	71 (63–90)	<0.01	72 (63.8–91.5)	79 (70–91.5)	0.354
LVEF, %	58 (50–65.5)	60 (52–67)	0.43	*	*	*
TTE–LA volume index, ml/m^2^	40 (30–49.45)	43 (34–53)	0.2	*	*	*
Systolic pulmonary artery pressure, mmHg	28 (25–32)	30 (26.5–34)	0.18	*	*	*
Per-procedural LAIV excluding LAA, ml	140 (116–152)	120 (100–140)	0.06	139 (120–150)	120 (100–140)	0.070
Pre-procedural LAIVI excluding LAA, ml m^2^	63 (55–71)	63 (53–83)	0.71	64 (59–70)	63 (58–69)	0.925

ACEi/ARB, angiotensin-converting enzyme inhibitor/angiotensin II receptor blocker; LVEF, left ventricle ejection fraction; OSA, obstructive sleep apnea.

Data are presented as a value (with percentage) for categorical variables, and median (25th−75th percentile) or mean ± SD for quantitative variables. A two-tailed *p*-value of <0.05 was considered significant. Time to treatment = time from first clinical diagnosis of AF to ablation procedure.

### 3.2. Gender differences in bipolar voltage mapping

The median number of total mapping points collected per map was 944 (573–1,640) in the whole unmatched cohort and was similar for both sexes (*p* = 0.52). The total mapping points per map collected in men and women were, respectively, 944 (560–1,669) and 1,144 (586–1,664) (Supplementary Table S1). The median global LA [1.2 (0.8–1.9) mV vs. 2.4 (1.5–2.7) mV, *p* < 0.01] and each regional (*p* < 0.01) bipolar voltage amplitudes were lower in women in the unmatched cohort (Table 2). After PS matching, the median global LA [1.1 (0.8–1.5) mV vs. 1.84 (1.35–2.66) mV, *p* = 0.002] and each regional (*p* < 0.05) bipolar voltage amplitudes were lower in women except for the lateral wall at the limit of significance (*p* = 0.059).

**Table 2:** Median regional distribution of bipolar voltage amplitudes according to gender before and after PS matching.

	Before PS matching			PS-matched		
	Men (*n* = 74)	Women (*n* = 41)	*p-*value	Men (*n* = 28)	Women (*n* = 28)	*p*-value
Median and regional bipolar voltage amplitude, mV						
Global left atrium	2.4 (1.5–2.7)	1.2 (0.8–1.9)	<0.01	1.8 (1.4–2.7)	1.1 (0.8–1.5)	0.002
Anterior	1.9 (1.4–2.6)	1.1 (0.7–1.5)	<0.01	1.8 (1.1–2.1)	0.9 (0.7–1.3)	0.001
Septal	1.7 (1.2–2.4)	0.9 (0.6–1.3)	<0.01	1.5 (0.9–2.1)	0.9 (0.6–1.1)	0.007
Posterior	2.3 (1.7–3.1)	1.1 (0.6–1.9)	<0.01	2.2 (1.4–3.5)	1.0 (0.6–1.7)	0.002
Inferior	2.0 (1.2–2.7)	1.3 (0.8–1.9)	<0.01	1.9 (1.1–2.8)	1.3 (0.8–1.8)	0.042
Lateral	2.3 (1.4–3.1)	1.4 (0.8–2.3)	<0.01	2.1 (1.2–2.7)	1.3 (0.6–2.1)	0.059
Left atrial appendage	3.0 (2.1–3.9)	1.8 (1.2–2.8)	<0.01	2.5 (2.1–3.5)	1.6 (1.2–2.7)	0.011

Data are presented as a median (25th−75th percentile). A two-tailed *p*-value of <0.05 was considered significant.

**Figure 1:**
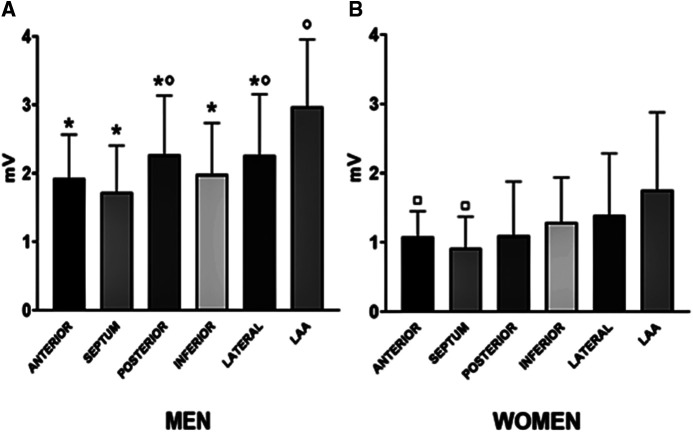
Median regional distribution of bipolar voltage amplitudes according to gender: men (**A**) and women (**B**). *p < 0.05 indicated the statistical difference as compared with the LAA region among men. °*p *< 0.05 indicated the statistical difference as compared with the septum region among men. ^□^*p* < 0.05 indicated the statistical difference as compared with the LAA region among women.

In the unmatched cohort, LAA bipolar voltage in men was higher compared with other atrial regions (Figure 1A). The septum highlighted the lowest voltage values [1.7 (1.2–2.4) mV], and the differences reached a statistical and significant magnitude compared with LAA (*p* < 0.0001), the posterior wall (*p* = 0.004), and the lateral wall (*p* = 0.03).

For the unmatched cohort, in women, a homogenous voltage was observed across all regions but LAA (Figure 1B). Indeed, LAA bipolar voltage [1.8 (1.1–2.9) mV] was higher compared with the septum (*p* = 0.001) and anterior (*p* = 0.004) region.

**Figure 2:**
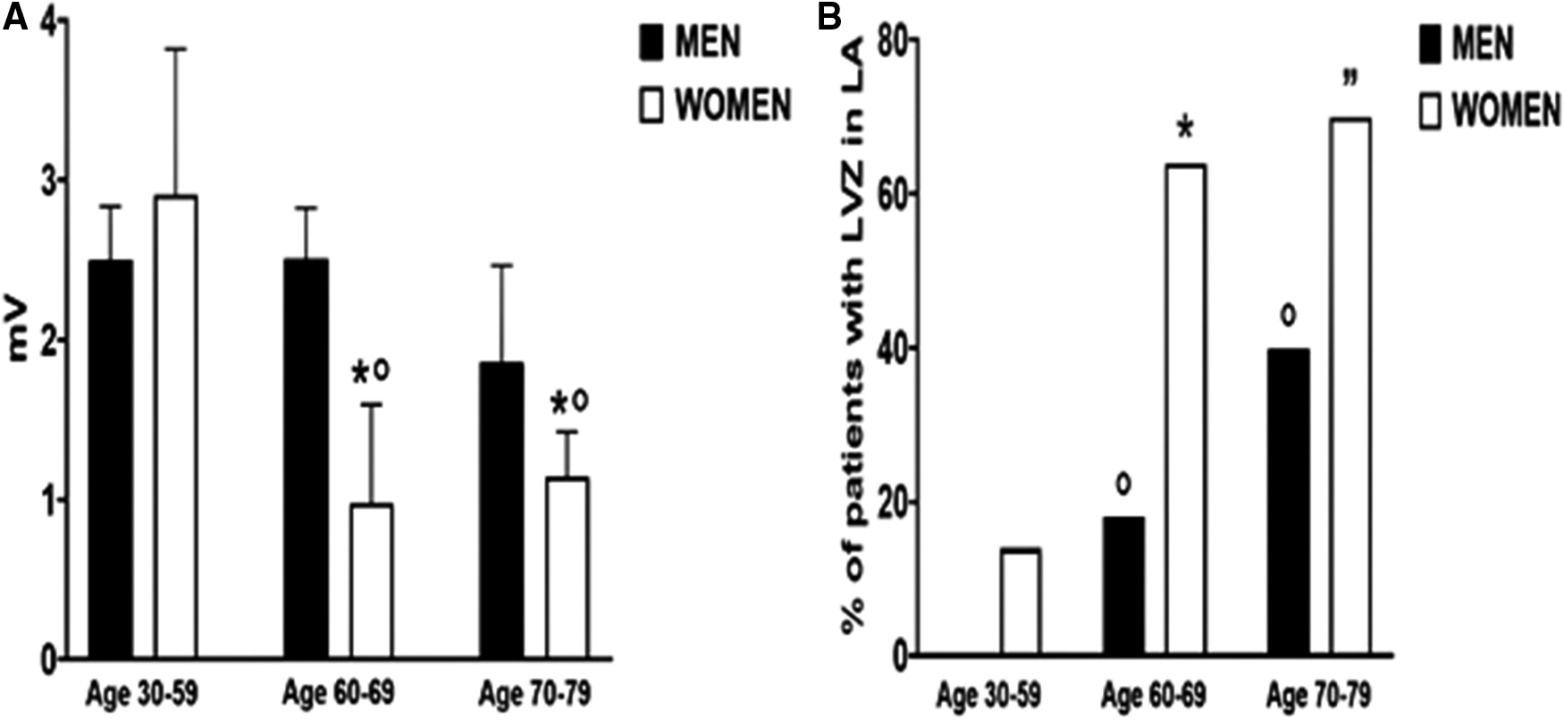
Median left atrial bipolar voltage amplitude (**A**) and low-voltage zones (**B**) according to gender and three different age groups (30–50, 60–69, and 70–79 years old. (**A**) *p < 0.05 indicated the statistical difference between men and women in the same age group. °*p* < 0.05 indicated the statistical difference compared with the 30–59 age group in women. (**B**) *p < 0.05 indicated the statistical difference between men and women in the same age group. °*p* < 0.05 indicated the statistical difference compared with the 30–59 age group in men. ”*p* < 0.05 indicated the statistical difference compared with the 30–59 age group in women.

The unmatched population in this study was divided into three groups according to their age: a younger age group (men *n* = 25 and women *n* = 7, aged 30–59 years old), a middle age group (men *n* = 34 and women *n* = 11, aged 60–69 years old), and an older age group (men *n* = 15 and women *n* = 23, aged 70–79 years old). LA voltage was similar in both sexes (*p* = 0.99) in the younger age group (Figure 2A). The middle age group (*p* = 0.009) and the older age group (*p* = 0.07) showed lower LA voltage in women.

Among women, LA voltage decreased significantly after 59 years of age [30–59 years old: 2.9 (1.3–3.8) mV vs. 1 (0.7–1.6) mV, *p* = 0.007] without difference between the middle age and the older age group (*p* = 0.99). In men, LA voltage remained stable across age groups and did not reach statistical significance between extreme age groups (*p* = 0.37).

### 3.2. Sex differences in low-voltage zones

The LVZ were evidenced in 30% of the whole unmatched cohort and were predominantly observed in the septum and the anterior and posterior walls (Table 3).

**Table 3:** LVZ extent and distribution according to gender before and after PS matching. ***Absence of data because statistical analysis is impossible to perform.

	Before PS matching			PS-matched		
	Men (*n* = 74)	Women (*n* = 41)	*p-*value	Men (*n* = 28)	Women (*n* = 28)	*p-*value
LVZ extent calculated as the percentage of LA surface area						
LVZ, *n* (%)	12 (16.2)	23 (56.1)	<0.01	7 (25.0)	18 (64.3)	0.007
No LVZ, *n* (%)	62 (83.8)	18 (43.9)	<0.01	21 (75.0)	10 (35.7)	0.007
Mild LVZ, *n* (%)	6 (8.11)	10 (25)	0.03	4 (14.3)	8 (28.6)	0.329
Moderate LVZ, *n* (%)	5 (6.76)	7 (17.5)	0.11	2 (7.1)	5 (17.9)	0.422
Severe LVZ, *n* (%)	1 (1.35)	5 (12.5)	0.02	1 (3.6)	5 (17.9)	0.193
LVZ surface (cm^2^) indexed to regional atrial surface (cm^2^), %						
LVZ/LA area	6.9 (2.1–21)	20 (8.1–33)	0.01	0.0 (0.0–4.6)	8.5 (0.0–28.1)	0.012
Anterior LVZ/anterior area	17.2 (8.8–29.5)	35.6 (18.8–52.4)	0.02	0.0 (0.0–7.8)	17.8 (0.0–43.0)	0.002
Septal LVZ/septal area	14.2 (7.8–26)	21.9 (13.9–42.9)	0.06	0.0 (0.0–6.1)	9.0 (0.0–37.5)	0.014
Posterior LVZ/posterior area	30.1 (18.1–45.8)	32.2 (15–40.4)	0.79	0.0 (0.0–0.0)	0.0 (0.0–6.0)	0.352
Inferior LVZ/inferior area	***	***	***	0.0 (0.0–0.0)	0.0 (0.0–1.5)	0.587
Lateral LVZ/lateral area	***	***	***	0.0 (0.0–0.0)	0.0 (0.0–0.0)	0.146
LAA LVZ/LAA area	***	***	***	0.0 (0.0–0.0)	0.0 (0.0–0.0)	0.036
Number of regional LVZ, *n* (%)						
Anterior	12 (16.2)	23 (56.1)	<0.01	8 (28.6)	17 (60.7)	0.031
Septal	16 (21.6)	16 (39)	0.08	9 (32.1)	12 (42.9)	0.582
Posterior	8 (10.8)	10 (24.4)	0.1	4 (14.3)	6 (21.4)	0.729
Inferior	1 (1.4)	5 (12.2)	0.02	1 (3.6)	4 (14.3)	0.352
Lateral	1 (1.4)	1 (2.4)	1	1 (3.6)	1 (3.6)	1.000
LAA	0 (0)	5 (12.2)	<0.01	0 (0.0)	3 (10.7)	0.236

All data are presented as a value (percentage) for categorical variables or median (25th–75th percentile) for quantitative variables. A two-tailed *p*-value of <0.05 was considered significant.

In the unmatched cohort, LVZ were more frequently evidenced in women (*p* < 0.01) (Table 3). Higher LVZ extent indexed to LA surface (*p* = 0.01), LVZ area ranking from 5% to 20% of LA (*p* = 0.03), and anterior localization (*p* < 0.01) were more frequently observed in women. The P-LAA duration was also significantly prolonged in women compared with men [104 (91–129) vs. 90 (81–100), *p* < 0.01] attesting to an anterior remodeling.

After PS matching, LVZ were more frequently evidenced in women (*p* = 0.007) (Table 3). Higher LVZ extent indexed to LA surface (*p* = 0.012) and especially anterior localization (*p* = 0.002) were more frequently observed in women.

There was no difference in LVZ between men and women until 59 years of age (Figure 2B), but its incidence dramatically increased after 60 years of age compared with male counterparts (64% vs. 17.7%, *p* = 0.007). LVZ increased in men in the older age group compared with the younger age group (40% vs. 0%, *p* = 0.001).

### 3.3. Ablation results in the unmatched cohort

All PVs were successfully isolated during the CA procedure (Supplementary Table S1). PVI alone was performed in 71 (61.7%) patients of the overall cohort, while the remaining 44 patients (38.3%) had an additional LVZ-guided substrate ablation.

PVI alone was more frequently performed in men [55 (74.3%) vs. 16 (39%), *p* < 0.01) compared with women. There was no difference between the two groups for cavo-tricuspid isthmus (CTI) ablation [14 (19%) vs. 6 (14.6%), *p* = 0.75]. The total RF duration (*p* = 0.02) and the fluoroscopy time (*p* < 0.01) were higher in women compared with men.

### 3.4. Clinical outcome after persistent AF catheter ablation

**Figure 3:**
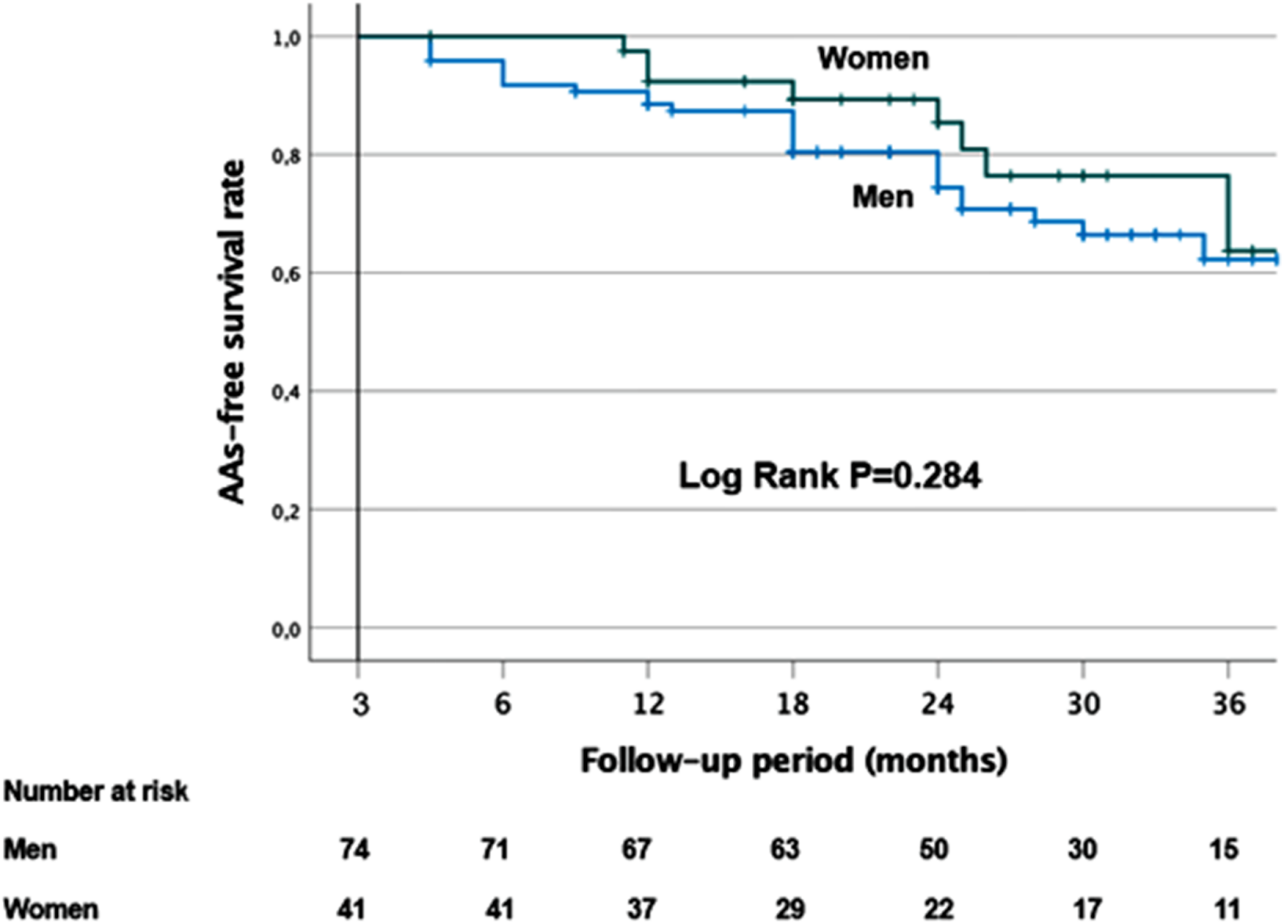
Kaplan–Meier survival curves showing the cumulative AF/AT recurrence-free survival rates according to gender.

After a follow-up period of 37.2 (33.9–40.5) months in this unmatched cohort, AAs recurrence could be observed in 12 out of 115 (10.4%) patients after one single procedure. The Kaplan–Meier survival curves are shown in Figure 3. There was no difference in the AA-free survival rate after one procedure between women and men (log-rank test, *p *= 0.284). In total, 61.8% of women and 60.8% of men remained free of AF/AT after 36 months (Figure 3). In the whole cohort, AADs were discontinued in 68% (78/115) of patients.

Among patients without AA recurrence at 12 months, AADs were discontinued in 69% (71/103) of the cohort, 56.4% (22/39) in women and 76.5% (49/64) in men (*p* = 0.05).

There was also no difference in the AA-free survival rate after one procedure between women who had PVI alone and those with additional LVZ ablation (log-rank test, *p *= 0.875). In total, 61% of women with PVI alone and 63% of women with additional LVZ ablation remained free of AF/AT after 36 months (Supplementary Figure S4).

### 3.5. Predictive factors of AAs recurrence

To evaluate the predictors of AAs recurrence after CA in this unmatched cohort, univariate and multivariate analyses were performed in the whole cohort. In the total population, only the *P*-wave duration (PWD) (*p* = 0.013) was a predictor of AF recurrence after CA (Table 4).

**Table 4:** Predictors of atrial arrhythmias recurrence after AF ablation in the global population.

	Univariate analysis			Multivariate analysis		
	OR	95% CI	*p-*value	OR	95% CI	*p*-value
Female gender	3.047	0.634–14.638	0.164			
Age	1.004	0.939–1.073	0.907			
AF duration	0.736	0.150–3.610	0.706			
Time to treatment	1.000	1.000–1.001	0.586			
BMI	0.944	0.837–1.066	0.354			
Dyslipidemia	0.986	0.277–3.504	0.982			
Hypertension	1.641	0.494–5.446	0.418			
Smoking	0.315	0.084–1.185	0.087	0.227	0.046–1.116	0.068
OSA	0.322	0.095–1.092	0.069	0.327	0.075–1.429	0.137
Coronary artery disease	0.239	0.062–0.926	0.038	0.416	0.083–2.091	0.287
Sinus node dysfunction	0.421	0.078–2.261	0.313			
ACEi/ARB	0.855	0.241–3.031	0.809			
Aldosterone receptor antagonist	0.212	0.061–0.732	0.014	0.222	0.046–1.067	0.060
eGFR	1.000	0.966–1.035	0.994			
LAIVI	1.016	0.979–1.053	0.402			
*P*-wave duration	1.041	1.010–1.073	0.009	1.053	1.011–1.096	0.013
LA bipolar voltage	1.004	0.538–1.874	0.990			
Presence of LVZ	0.861	0.241–3.072	0.818			
Only PVI	1.172	0.348–3.950	0.798			

Data are presented as an odd ratio with 95% CI. A two-tailed *p*-value of <0.05 was considered significant. Time to treatment = time from first clinical diagnosis of AF to ablation procedure.

### 3.6. Predictors of low-voltage zone

To evaluate the predictors of LVZ in this unmatched cohort, univariate and multivariate analyses were performed in the whole population and according to gender. In the whole population, the female sex (*p* = 0.001), age (*p* = 0.002), and LA indexed volume (*p* = 0.001) were identified as the predictors of LVZ (Table 5).

**Table 5:** Predictors of the presence of low-voltage zone in the global population.

	Univariate analysis			Multivariate analysis		
	OR	95% CI	*p-*value	OR	95% CI	*p-*value
Female gender	0.151	0.063–0.363	0.0001	0.124	0.035–0.436	0.001
Age	1.205	1.112–1.305	0.0001	1,158	1.056–1.271	0.002
AF duration	1.19	0.751–1.875	0.46			
Time to treatment	1	1.000–1.001	0.087	1,000	1.000–1.001	0.083
BMI	0.882	0.805–0.965	0.007	0.918	0.823–1.025	0.128
Dyslipidemia	0.769	0.334–1.769	0.537			
Hypertension	0.514	0.218–1.211	0.128			
Diabetes mellitus	0.848	0.309–2,328	0.75			
Smoking	1.644	0.500–5.406	0.413			
OSA	2.135	0.861–5,295	0.102			
Coronary artery disease	0.613	0.200–1.877	0.391			
Sinus node dysfunction	0.400	0.108–1.482	0.17			
ACEi/ARB	1.472	0.652–3.323	0.352			
Aldosterone receptor antagonist	0.844	0.323–2,205	0.729			
eGFR	0.962	0.938–0.985	0.002	1.001	0.961–1.043	0.946
LAIVI	1.086	1.047–1.127	0.0001	1.097	1.041–1.156	0.001
*P*-wave duration	1.002	0.985–1.019	0.822			

Data are presented as an odd ratio with 95% CI. A two-tailed *p*-value of <0.05 was considered significant*.* Time to treatment = time from first clinical diagnosis of AF to ablation procedure.

Only LA indexed volume (*p* = 0.048) was identified as a predictor for LVZ in women (Supplementary Table S2). In men, the age (*p* = 0.01) and LA indexed volume (*p* = 0.007) were predictive of LVZ (Supplementary Table S2).

## 4. Discussion

The current report demonstrated a strong relationship between the female gender and atrial substrate remodeling, using electro-anatomical mapping. Both global and regional LA bipolar voltage appeared twice lower in women, even though the LA indexed volumes were similar in both sexes. We found that LVZ occurred earlier in women. Moreover, the female gender was significantly associated with a higher incidence and a greater extent of LVZ, especially in the LA anterior wall, attested by an increased P-LAA duration. The female gender, age, and LA indexed volume were the predictors of LVZ in persistent AF patients. Despite a significant difference in the LA remodeling extent according to gender, LVZ-guided ablation outcome was similar and favorable in both sexes. The PWD was the sole predictor of AAs recurrence in the whole cohort.

### 4.1. Gender differences in LA bipolar voltage

Several studies demonstrated lower LA voltage amplitude in AF compared with SR across all LA regions (19). Conversely, only scarce data exist in AF ablation patients regarding bipolar voltage in SR according to gender. Only Wong et al. (3) demonstrated that global mean voltage was significantly lower in women compared with men with 50% paroxysmal AF.

Our study is among the first reports to highlight that bipolar voltage was twice lower in both LA and each atrial region in women compared with men for a similar LA indexed volume. Kim et al. (12) also reported a lower LA bipolar voltage associated with higher periatrial adiposity in postmenopausal women compared with men but in smaller magnitude. This may be explained at least in part by the lower rate of persistent AF (around 40%) that is typically associated with higher LA remodeling. Lower LA bipolar voltage that occurred prematurely in women is highly suggestive of a time-dependent association and a possible hormonal-dependent and postmenopausal shift of sex-related LA remodeling.

### 4.2. LVZ and sex

Controversial results remain about gender-related impact to clinical outcomes after AF ablation (20). A recent large-scale multicenter registry reported that women presented higher arrhythmia recurrences, whereas PV reconnections were lower in women. These data demonstrated that non-PV arrhythmogenicity may play a more important role in women than in men (21).

Wong et al. (3) demonstrated gender-related differences in atrial substrate remodeling characterized by lower voltage, reduced conduction velocity and increased complex fractionated signals, and higher arrhythmia recurrences following PVI in women.

LA remodeling and LVZ in particular could therefore explain such gender differences in AF recurrence rates.

Consistent with our findings, LVZ has been reported to be related to both AF recurrence after ablation (6) and the female gender (10, 11). Women is therefore a challenging subgroup of persistent AF patients with a distribution of LA remodeling variable but more pronounced anteriorly as observed in our study population. Our anatomic distribution shares similarities to the report of Schreiber et al. (11), in which women seemed to show fibrotic atrial cardiomyopathy more often than men and particularly anteriorly. Our results are also consistent with the recent studies on LGE-MRI imaging that evidenced higher extent of atrial fibrosis in AF women (4). All together, these data suggest that the LVZ extent in women could contribute to lower the success rates of AF ablation.

Consistent with the previous finding (17), LVZ were more frequently localized in the septum and the anterior and posterior walls in both sexes, and such heterogeneous distribution may be explained at least in part by LA regional differences in wall stress (22). The LVZ progression within human atrium is still under study. Both enlarged LA volume and continuous atrial stretch are known key contributors to atrial electrical remodeling (23). We found that LA indexed volume in the whole cohort was predictive of LVZ presence. However, the LA indexed volume in our cohort was similar in both sexes and thus could not explain this difference in LA remodeling. Quantifying the effects of other proposed mechanisms such as age, hormones, and external stretch is an important research topic that needs more attention to shed light on the “female paradox” of more extensive LA remodeling.

The external structure may also influence the LVZ progression and more specifically the contact between LA and the ascending thoracic aorta causing external stress, which may lead to the progression of the anterior wall remodeling (24). In the present study, women presented more frequent and larger anterior LVZ than men, whereas there was no difference in the LVZ incidence and size in other atrial regions. So far, no data on external structure comparing men and women have been described.

Interestingly, we evidenced that the LVZ presence and the decrease in LA bipolar voltage occurred earlier in women, which could explain gender differences in the time course of fibrosis remodeling. Women usually develop AF after menopause (2), and hormonal changes may be the key mechanism contributing to the progression of LA fibrosis and remodeling in postmenopausal women. It has already been evidenced that estrogens may play a pivotal role in modulating electrophysiological properties (25). However, their involvement in the AF pathogenesis in women still remains to be elucidated. Other factors such as genetic and gender differences in protein expression could contribute to increased atrial fibrosis in women. An increase in the plasma concentration of the inflammatory marker C-reactive protein and of fibroblast growth factor-23 was observed in women (26, 27). Lately, gender differences in fibrosis remodeling were attributed to the inherent differential expression of fibrosis-related genes and proteins. Indeed, Li et al. (28) showed that the TGFβ/Smad 3 pathway was upregulated in women with long standing persistent AF in case of mitral valve disease. There is no data in case of lone early persistent AF as in our population. Moreover, increasing evidence suggested that epicardial atrial fat is associated with AF, inducing promotion of local inflammation and atrial structural remodeling (29). LA remodeling associated with epicardial fat could be different between men and women with AF, even though they are exposed to the same clinical conditions. Huo et al. (17) showed that the female gender and age were predictive factors of LVZ and that postmenopausal women had a greater degree of periatrial adiposity, which could be linked to the decreased bipolar LA voltage.

### 4.3. Favoring factors of LVZ

Some authors have reported an association between LVZ and age (10, 17). In the present study, a gradual increase in LVZ with age was observed only in men, whereas LVZ appeared earlier and extensively in women. The multivariate analysis evidenced that age was a strong predictor of LVZ in the whole population and in men subgroup. In women, only the LA indexed volume was evidenced as a predictive factor of LVZ, possibly linked to the complex interplay between age, menopause, BMI, epicardial fat, and cardiovascular risk factors, all involved in LA remodeling.

Matsuda et al. (30) reported an association between renal dysfunction and LVZ presence in patients undergoing AF ablation as well as a higher AF recurrence rate in patients with chronic kidney disease, defined as eGFR <60 ml/min/1.73 m^2^. Renal dysfunction associated with an activation of pro-inflammatory and pro-fibrotic pathways at the cellular level was found to be a predictor for the presence of LVZ and recurrence after AF ablation (30). In our study, women presented a mild altered kidney function compared with men, but eGFR remained superior to 60 ml/min/1.73 m^2^, and such observation could not be done.

### 4.4. LVZ-guided ablation results and predictor of AAs recurrence

Several studies have shown that women had higher AF recurrence rate after initial and multiple CA procedures (3, 21).

This gender difference in the recurrence rate could be due to increased non-PV triggers and more AF substrate in women. LVZ presence is known to be a powerful predictor of AF recurrence after PVI alone (9). Tanaka et al. (21) observed that 58% of women and 65% of men were free from AAs recurrence 1 year after CA for persistent AF. However, in our study, we reported a better outcome without difference between genders, 62% of women and 61% of men free from AAs recurrence at 36 months with 56% of women off drugs vs. 77% of men. PVI alone was achieved only in 39% of women and 74% of men, explaining the difference in total RF duration and fluoroscopy time between men and women. Our study is among the first to assess the outcome of LVZ-guided RF ablation according to gender with similar results compared with men, which are encouraging for women prognosis. With this voltage-guided strategy, LVZ presence may not be anymore considered as a predictor of AF recurrence. Taking into account the number of patients who are still under AADs during the follow-up and the retrospective observational study design, these results must be carefully interpreted, and further randomized studies are needed to confirm them.

We also found that PWD was the sole predictive factor of AAs recurrence after a single procedure of AF ablation in the whole cohort. A prolonged PWD corresponds to inter- and intra-atrial abnormal conduction delay. It was also correlated with atrial electro-anatomical delay assessed by tissue Doppler echocardiography (31). A relationship between PWD and AAs recurrence had already been reported (32). Moreover, few studies underlined an association between prolonged PWD and LA low-voltage substrate in both persistent and paroxysmal AF patients (33, 34).

### 4.5. Study limitations

Our study was a single-center retrospective observational study. Therefore, one potential limitation is the cohort size. Including a greater number of patients particularly women would help to validate our findings. Moreover, LA bipolar voltage maps were obtained without multipolar catheter, which would have provided more rapid and extensive maps with higher resolution of LVZ. In addition, discontinuation of AADs is variable and handled by the cardiologist of the patient. It could have influenced the ablation results. Further studies are mandatory to evaluate the long-term follow-up after a voltage-guided AF ablation according to gender.

## 5. Conclusion

This study reports a strong relationship between the female gender and atrial substrate remodeling, using electro-anatomical mapping. The female gender and LA indexed volume were both independent predictors of left atrial LVZ in persistent AF patients. The global and regional LA bipolar voltage was lower in women. We found that the female gender was significantly associated with a higher incidence, earlier occurrence, and greater extent of LVZ compared with men. Despite female-specific characteristics in atrial remodeling, LVZ-guided ablation may improve the AF ablation outcome in women.

## Data Availability

The original contributions presented in the study are included in the article/Supplementary Material, further inquiries can be directed to the corresponding author.
