# Supplementary material for: Impact of gender on left atrial low-voltage zones in patients with persistent atrial fibrillation: results of a voltage-guided ablation
Source: Front Cardiovasc Med. 2023 Aug 24;10:1229345. doi: 10.3389/fcvm.2023.1229345 (PMC10484507; doi:10.3389/fcvm.2023.1229345)
Supplement: Supplementary file 1 [file Table1.doc]

**Table S1.** Procedural data according to gender.

|  | **Men (n=74)** | **Women (n=41)** | **P value** |
| --- | --- | --- | --- |
| Fluoroscopic time, min | 20.2 ± 4.9 | 37 ± 10 | **<0.01** |
| Median total mapping points per map | 944 [560-1669] | 1144 [586-1664] | 0.52 |
| Total RF duration, min | 30.8 ± 9.9 | 35.8 ± 12.3 | **0.02** |
| AF at admission before procedure | 20 (27%) | 16 (39%) | 0.26 |
| Only PVI | 55 (74%) | 16 (39%) | **<0.01** |
| CTI ablation before or during procedure | 14 (19%) | 6 (15%) | 0.75 |

Data are presented as a value (with percentage) for categorical variables, mean (with standard deviations) and median (25^th^–75^th^ percentile) for quantitative variables. A two-tailed p value<0.05 was considered significant.

**Abbreviations:** *Min, minute; RF, radiofrequency; AF, atrial fibrillation; PVI, pulmonary vein isolation; CTI, cavo-tricuspid isthmus.*

**Table S2.** Predictors of the presence of low-voltage zone according to gender

|  | **MEN** | | | | | | | **WOMEN** | | | | | |
| --- | --- | --- | --- | --- | --- | --- | --- | --- | --- | --- | --- | --- | --- |
|  | **Univariate Analysis** | | | **Multivariate Analysis** | | | | **Univariate Analysis** | | | **Multivariate Analysis** | | |
|  | OR | 95% CI | P Value | OR | 95% CI | P value | OR | | 95% CI | P Value | OR | 95% CI | P Value |
| Age | 1.24 | 1.091-1.403 | 0.001 | 1.23 | 1.04-1.44 | 0.01 | | 1.136 | 1.026-1.258 | 0.014 | 1.061 | 0.922-1.221 | 0.408 |
| AF duration | 1.05 | 0.536-2.057 | 0.89 |  |  |  | | 0.950 | 0.184-4.912 | 0.951 |  | | |
| Time to treatment | 1 | 1.000-1.001 | 0.25 |  |  |  | | 1.001 | 1.000-1.001 | 0.076 |  |  |  |
| BMI | 0.94 | 0.781-1.128 | 0.5 |  |  |  | | 0.837 | 0.746-0.939 | 0.002 | 0.879 | 0.771-1.002 | 0.054 |
| Hyperlipidemia | 0.98 | 0.263-3.619 | 0.97 |  |  |  | | 0.444 | 0.111-1.787 | 0.253 |  | | |
| Hypertension | 1.54 | 0.420-5.667 | 0.51 |  |  |  | | 0.441 | 0.118-1.644 | 0.223 |  |  |  |
| Diabetes mellitus | 1.18 | 0.221-6.285 | 0.85 |  |  |  | | 1.385 | 0.331-5.787 | 0.656 |  |  |  |
| Smoking | 0.83 | 0.161-4.312 | 0.83 |  |  |  | | 1.312 | 0.166-10.350 | 0.796 |  |  |  |
| OSAS | 0.65 | 0.159-2.662 | 0.55 |  |  |  | | 4.500 | 1.156-17.510 | 0.03 | 1.357 | 0.205-8.959 | 0.751 |
| Paroxysmal AF history | 1.05 | 0.282-3.903 | 0.94 |  |  |  | | 0.184 | 0.046-0.738 | 0.017 | 1.651 | 0.210-12.997 | 0.634 |
| ACEI/ARB | 0.57 | 0.161-2.043 | 0.39 |  |  |  | | 0.769 | 0.223-2.654 | 0.678 |  | | |
| Aldosterone receptor antagonist | 0.83 | 0.161-4.312 | 0.83 |  |  |  | | 0.810 | 0.190-3.449 | 0.775 |  |  |  |
| eGFR | 0.94 | 0.902-0.985 | 0.01 | 0.97 | 0.9-1.04 | 0.41 | | 0.992 | 0.960-1.025 | 0.637 |  |  |  |
| LAIVI | 1.11 | 1.042-1.182 | 0.001 | 1.1 | 1.026-1.181 | 0.007 | | 1.113 | 1.029-1.203 | 0.007 | 1.107 | 1.001-1.225 | **0.048** |

Data are presented as an odd ratio with 95% CI. A two-tailed p value<0.05 was considered significant.

Time to treatment = time from first clinical diagnosis of AF to ablation procedure.

**Abbreviations:** *ACEI/ARB*, Angiotensin-converting enzyme inhibitor/angiotensin receptor blocker; *ATE, Arterial thromboembolism; AF, atrial fibrillation; BMI, body mass index;* *CI, confidence interval;* eGFR, estimated glomerular filtration rate; LAIVI, left atrial intracavitary volume index; *OR, odds ratio; OSA, obstructive sleep* apnea
