# Supplementary figures and images for: Impact of gender on left atrial low-voltage zones in patients with persistent atrial fibrillation: results of a voltage-guided ablation
Source: Front Cardiovasc Med. 2023 Aug 24;10:1229345. doi: 10.3389/fcvm.2023.1229345 (PMC10484507; doi:10.3389/fcvm.2023.1229345)

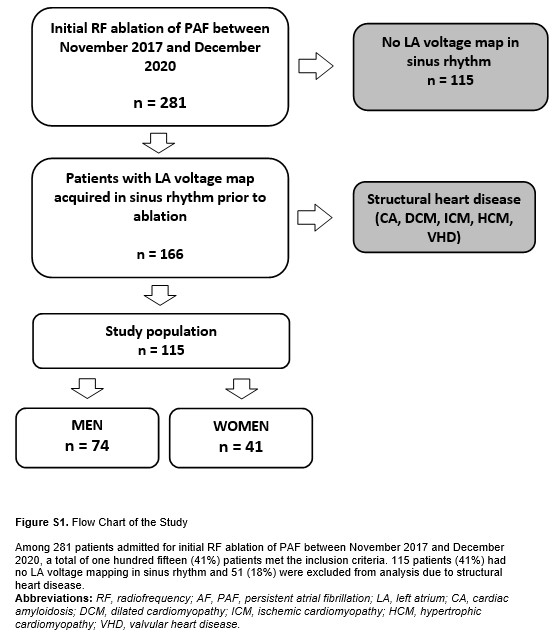

Supplement: Supplementary file 2 [file Image1.jpeg]

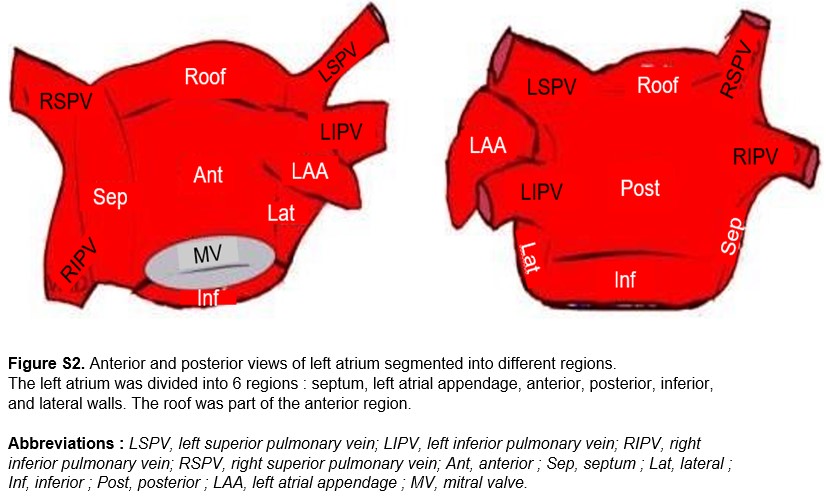

Supplement: Supplementary file 3 [file Image2.jpeg]

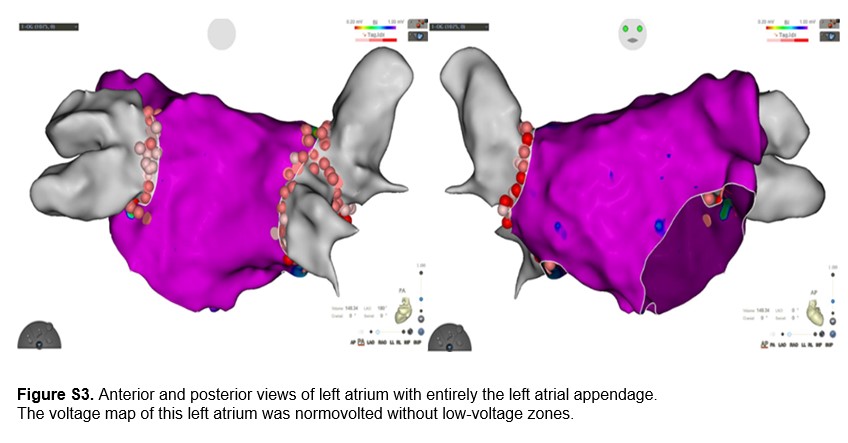

Supplement: Supplementary file 4 [file Image3.jpeg]

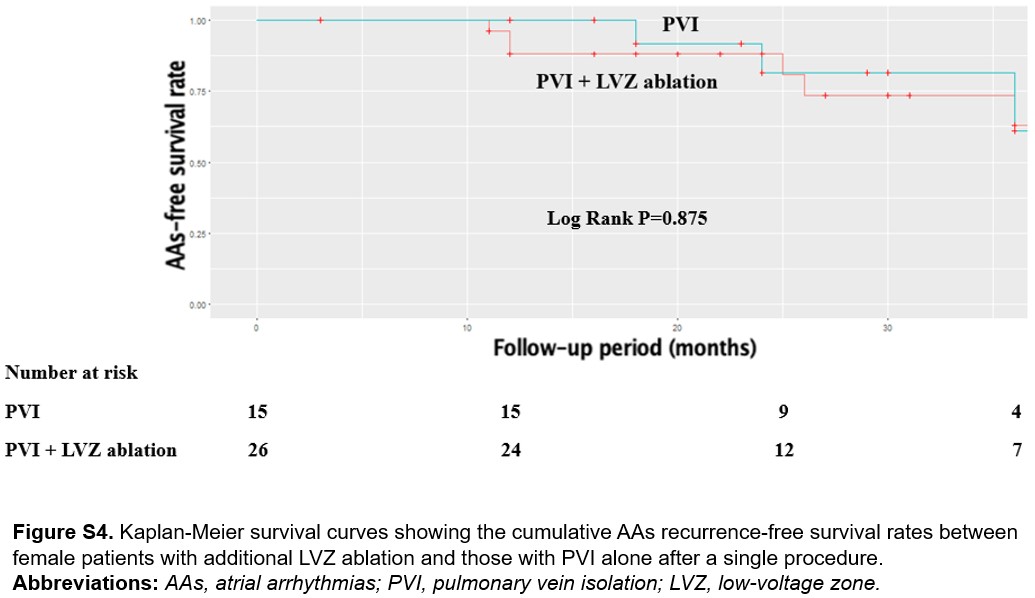

Supplement: Supplementary file 5 [file Image4.jpeg]
